# Functionalized graphene oxide-based lamellar membranes for organic solvent nanofiltration applications

**DOI:** 10.1039/d3ra00223c

**Published:** 2023-04-25

**Authors:** Ashique Hussain Jatoi, Kyung Hwan Kim, Muhammad Ali Khan, Fida Hussain Memon, Muzaffar Iqbal, Dahar Janwery, Shah Nawaz Phulpoto, Anupama Samantasinghar, Kyung Hyun Choi, Khalid Hussain Thebo

**Affiliations:** a Department of Chemistry, Shaheed Benazir Bhutto University Shaheed Benazirabad 67480 Pakistan; b BioSpero, Inc. Jeju Republic of Korea; c Institute of Chemical Sciences, Bahauddin Zakariya University Multan 60800 Pakistan; d Department of Mechatronics Engineering, Jeju National University Jeju 63243 Republic of Korea amm@jejunu.ac.kr; e Department of Electrical Engineering, Sukkur IBA University Sukkur 65200 Pakistan; f Department of Chemistry, Faculty of Physical and Applied Sciences, The University of Haripur KPK 22620 Pakistan; g National Centre of Excellence in Analytical Chemistry, University of Sindh Jamshoro Pakistan; h Department of Molecular Biology & Genetics, Shaheed Benazir University Shaheed Benazirabad 67480 Pakistan; i Institute of Metal Research, Chinese Academy of Sciences (CAS) Shenyang 110016 China Khalidthebo@yahoo.com

## Abstract

In this study, two-dimensional graphene oxide-based novel membranes were fabricated by modifying the surface of graphene oxide nanosheets with six-armed poly(ethylene glycol) (PEG) at room conditions. The as-modified PEGylated graphene oxide (PGO) membranes with unique layered structures and large interlayer spacing (∼1.12 nm) were utilized for organic solvent nanofiltration applications. The as-prepared 350 nm-thick PGO membrane offers a superior separation (>99%) against evans blue, methylene blue and rhodamine B dyes along with high methanol permeance ∼ 155 ± 10 L m^−2^ h^−1^, which is 10–100 times high compared to pristine GO membranes. Additionally, these membranes are stable for up to 20 days in organic solvent. Hence the results suggested that the as-synthesized PGO membranes with superior separation efficiency for dye molecules in organic solvent can be used in future for organic solvent nanofiltration application.

## Abstract 2

In this study, two-dimensional graphene oxide-based novel membranes were fabricated by modifying the surface of graphene oxide nanosheets with six-armed poly(ethylene glycol) (PEG) at room conditions and used for organic solvent nanofiltration applications.

## Graphical abstract

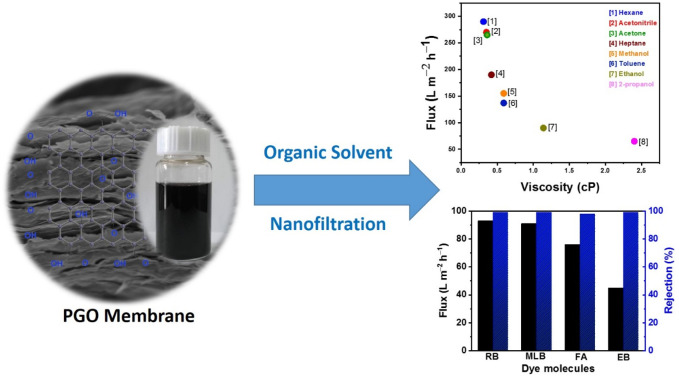

## 1. Introduction

Membrane-based technology has played a significant role in gas purification, water desalination, fuel cells, catalysis, hemofiltration, bioprocessing, pervaporation and other industrial separation processes.^1–11^ Among them, organic solvent nanofiltration (OSN) is the most promising technology used for separation and purification in organic solvents and is commonly utilized for chiral separation, catalyst recovery, ionic liquid separation, solvent recycling, *etc.*^12^ So far, several OSN membrane based on inorganic and organic (polymeric) materials have been developed and tested.^13^ Organic (polymeric) membranes are generally made up of polyimide (PI) and have advantages of ease of fabrication and low price, however these are limited in terms of flux, and separation efficiency.^13^ Inorganic membranes have superior stability in organic solvents; but the large scale production of such membranes is complicated and more costly than polymeric membranes.^14^ Therefore, it is challenging task to develop novel OSN membranes with superior stability in various organic solvents along with high separation performance and permeance simultaneously.

Recently, GO membranes have drawn much attention from separation industries due to their unique layered structures, controlled pore size, ideal thickness, high mechanical strength and easy functionalization.^15–20^ Based on these properties, such membranes exhibited outstanding separation for small ions, isotopes, organic dyes, antibiotics and other bio-molecules.^21–28^ The GO nanosheets have the unique combination of hydrophobic and hydrophilic domains along with hydroxyl, epoxide and carboxyl groups on its basal plane and edges.^29–31^ Such functional groups can attract water molecules and swell in aqueous environment. Therefore, these membranes rapidly delaminated and were destroyed during wastewater separation and purification processes. In this regard, new avenues shall be investigated for GO-based membranes especially separation in organic solvents or media. To date, few studies have demonstrated the use of GO membranes in OSN application.^32–35^ Shi *et al.*^35^ tested GO based membranes with large nanochannels ∼ 0.98 nm for OSN applications. Due to large nanochannels, the membranes showed high permeance for acetone, pyrene and toluene solvents, while block large size molecules *i.e.* Lumogen Red 300. In addition, these membranes are mechanically strong in different organic solvents. In another study, Nair *et al.*^33^ demonstrated highly efficient GO laminates for OSN applications. Such membranes with uniform 2D capillaries exhibited >99.9% rejection of low molecular weights dye molecules in methanol. These studies demonstrated the high separation efficiency and permeability of different solvent through GO based membranes, however; the separation mechanism of these membranes in organic media has not yet been systematically explored.^35^

Here, we report a PGO membranes with variable thicknesses for OSN applications. In the present study, we have prepared novel separation membranes, in which PEG (6-armed and linear structures) was covalently introduced into the surface of GO nanosheets *via* simple cross-linking under normal conditions. Such PGO membranes showed a high flux for various organic solvents together with excellent separation efficiency (>99%) for several probe molecules. In addition, the PGO membrane is very stable in methanol for up to 20 days. Considering this excellent separation performance and chemical stability, we believe that these membranes will have exciting opportunities in organic separation and other OSN related applications.

## 2. Experiments

### 2.1. Materials

Natural graphite powders (45 μm) and other chemicals such as sulfuric acid (H_2_SO_4_, Aldrich, 95–98%), sodium nitrate (NaNO_3_, Aldrich, 99%), potassium permanganate (KMnO_4_, Aldrich, 99%) used for GO synthesis were purchased from Sigma-Aldrich and Alfa Aesar. Six-armed PEG (*M*_w_ = 6000) used for preparation of pegylation of GO nanosheets was of high purity. Polyethersulfone (PES) support with 0.45 μm pore size was procured from Beijing Wodun Technology Development Co., Ltd. China.

### 2.2. Preparation of GO nanosheets

The Hummer's methods was used to prepared GO sheets.^36^ Initially 2 g of graphite natural flakes (500–60 nm) was taken into round bottom flask. Then added 2 g of sodium nitrate and 96 mL of concentrated sulfuric and mixed together with continuously stirring up to 60 min. Further, potassium permanganate (12 g) was added slowly to reaction mixture at 0 °C with constant stirring to avoid over heating up to 30 min and then at 35 °C for 120 min. Further 280 mL of distilled water was added dropwise to solution to dilute mixture, followed by addition of 10 mL of 30% hydrogen peroxide solution with constant stirring up to 10 min to obtained graphite oxide dispersion. After that graphite oxide dispersion was collected with help of high speed centrifuge (6000 rpm at 20 min). The obtained product was washed several times with DI water and maintain pH near to 7. Furthermore, graphite oxide was exfoliate to GO suspension using sonication at 80 W for 10 min. The as-synthesized GO suspension was collected with help of centrifuge at 3000 rpm for 20 min to remove particles and multilayer flakes. Finally, GO suspension was dried and used for characterization and fabrication of membranes.

### 2.3. PEG modification of GO sheets and preparation of membranes

The 0.25 g of as-prepared GO sheets was dispersed into 100 mL of DI with help of sonication to get uniform dispersion. Further, 0.5 g of monochloroacetic acid (MCA) and 0.6 g of sodium hydroxide were added to GO dispersion with continuous sonication for 1 h and then put on stirring at 70 °C temperature for conversion of –OH groups of GO to –COOH groups. Furthermore, these –COOH groups of GO shall be neutralized by filtration process. During this process, the optical density of carboxylated functionalized GO should be maintained at 0.4 by diluting dispersion with DI water. After that 1.0 g of 6-arm poly(ethylene oxide) was added to dispersion of carboxylated GO with continuous sonication for 20 min. Then 1-(3-dimethylaminopropyl)-3-ethylcarbodimide hydrochloride (EDC) was added with constant stirring up to 12 h to complete reaction and the quenched by 2-mercaptoethanol. Finally, black precipitate of PGO was obtained and then separated with help of centrifuge (4000 rpm in phosphate-buffered saline) and dried for further use.

The PGO and pristine GO membranes were prepared by vacuum filtration method. First, the 0.25 g of dried PGO powder was dispersed into 100 mL of DI water with help of tip sonication to prepare stock solution. Then 10 mL of PGO dispersion was taken from stock solution and mix with 40 mL of DI. The obtained blend was filtered through vacuum filtration on nylon support (0.45 μm). After that PGO membrane was prepared and dried at room temperature up to 12 h. The thickness of PGO membranes were controlled by volume of blend (15 mL, 20 mL 25 mL, *etc.*) filtered through vacuum filtration assembly. Pristine GO membranes were also prepared by same method.

### 2.4. Characterization of materials

The structural morphology of membranes were measured with field-emission scanning electron microscopy (FE-SEM) at 10 kV using Nova NanoFESEM 430. The XRD spectra was recorded using X-ray powder diffraction (XRD) (D-MAX/2400) with Cu Kα radiation at scanning rate of 4° min^−1^. Fourier-transform infrared spectroscopy (FTIR) spectra was measured to confirm the chemical bonding between GO nanosheets and PEG molecule in range of 400 to 4000 cm^−1^ with help of Bruker Tensor 27 spectrometer. Further, the chemical composition of materials was confirmed with help of X-ray photoelectron spectrometer (ESCALAB250 XPS) using Al Kα radiation (150 W, spot size 500 nm). All spectra were calibrated to binding energy of adventitious carbon (284.8 eV). Ultra-violet visible spectrophotometer (Varian Carry 50) was used to collect the spectra of feed and permeate solution of dyes.

### 2.5. Solvent flux and rejection efficiency

The solvent flux and separation efficiency of dye molecules were measured with help of vacuum filtration assembly (effective area of 47 mm) at room temperature with 250 mL feed solution and using 1.0 bar pressure. First the organic solvent flux was recorded after every ten min up to obtained stable reading. Then 250 mL of feed solution of dye was filtered through PGO membrane to measure its efficiency. The rejection efficiency (*R*) and flux (*J*) of membranes were calculated to according to eqn (1) and (2) respectively.1
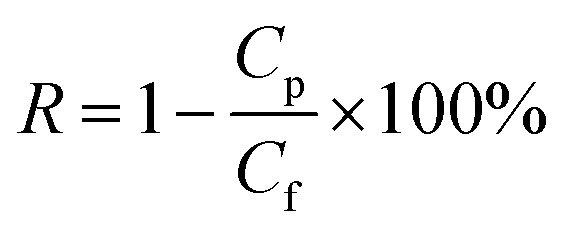
2
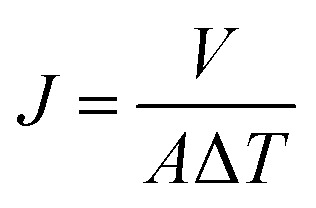
Here, “*C*_p_” and “*C*_f_” are concentrations of permeate and feed solution, while “*V*” is volume of permeate in liters, “*A*” is the area of membrane in m^2^, and “Δ*T*” is time obtained for permeate in h.

## 3. Result and discussions

### 3.1. Membrane preparation and characterization

**Fig. 1:**
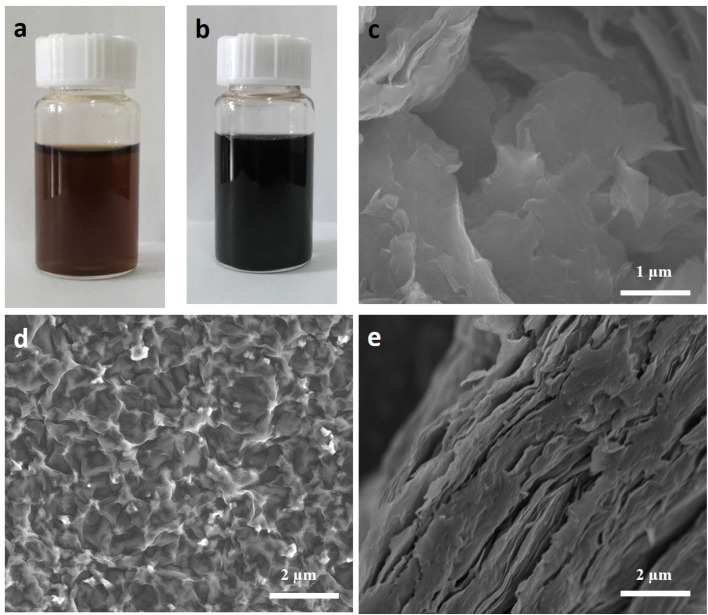
(a and b) Dispersion blend of GO (a) and PGO (b) respectively. (c and d) Surface SEM image of pristine GO and PGO membranes respectively. (e) Cross-sectional image of PGO membranes.

The pristine GO dispersion (Fig. 1a) was modified with 6-armed PEG molecule under mild conditions and PGO composite (Fig. 1b) is formed. Further, PGO membranes were prepared from PGO blend using vacuum filtration on nylon support. The surface morphology and structural difference between pristine GO and newly fabricated PGO membranes was characterized by SEM (Fig. 1c–e). The SEM studies showed the rough and irregular surface of pristine GO membranes as shown in Fig. 1c. However after modification, the PGO membrane indicated relatively smooth surface (Fig. 1d) compared to pristine GO membranes. While cross-sectional SEM study exhibited typical layered structures with large interlayer spacing of PGO membranes (Fig. 1e).

**Fig. 2:**
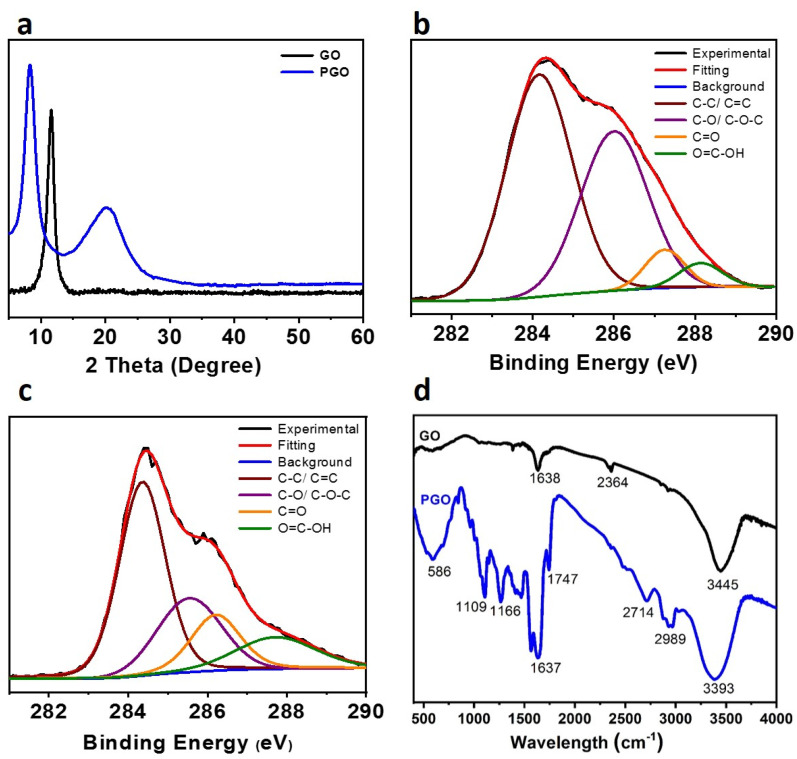
(a) The XRD pattern of GO and PGO membranes. (b and c) High resolution XPS spectra of carbon region (C 1s) of PGO (b) and GO (c) membranes respectively. (d) FTIR spectra of GO based membranes.

The XRD pattern of pristine GO and PGO membranes was collected to determine the interlayer distance between 2D sheets. The pristine GO membrane exhibited a strong diffraction peak at 10.9°, with interlayer distance of 0.75 nm as shown in Fig. 2a, which is dedicated due to oxygen containing functional groups and trapping of water molecules within 2D sheets. After modification of GO sheets with PEG molecule, the interlayer distance between 2D is increased up to 1.12 nm (2*θ* = 7.9°) as shown in Fig. 2a. This increased interlayer distance confirmed the successfully cross-linking of PEG molecule within GO sheets.

**Fig. 3:**
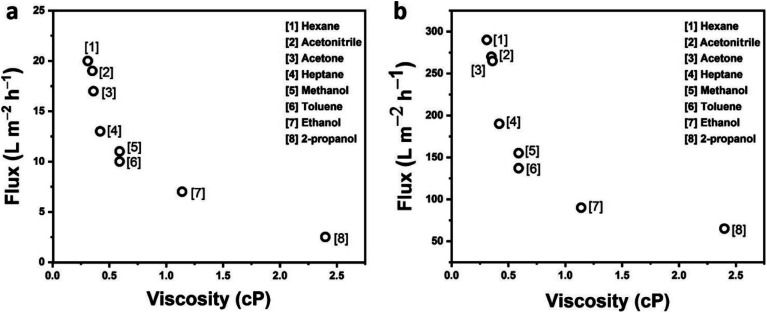
The flux of different solvents with variables viscosity through (a) pristine GO and (b) PGO membranes respectively.

Furthermore, functional groups characterization of pure GO and PGO membranes were carried out with help of XPS and FTIR studies (Fig. 3b–d). The four fitted C 1s deconvoluted curve of PGO membranes is shown in Fig. 3b. The Fig. 3b clearly showed binding energy of 284.6 eV, 285.8 eV, and 286.9 eV which represents the C–C/C=C, C–N and C=O respectively. While pure GO membranes showed similar XPS spectra to previous reported work (Fig. 3c).^28^ The peak observed at 284.2 eV represents the C=C/C–C, which is due to the aromatic ring of GO membranes. While peak at 286.2 eV showed the presence of 1,2 peroxide and alkoxyl functional groups. Furthermore, carbonyl (C=O) peak appears at 287.3 eV and epoxy (–C–O–C–) peak is confirmed at 288.7 eV. It is observed that O/C atomic ratio decreased from 0.44 to 0.31 for PGO membranes, although PEG is rich with oxygen functional groups. These results proved that the GO has been reduced during intercalation process, which is consistent with the color change mentioned above (Fig. 1b). The reduction is possibly due to thermal treatment during preparation process.

Further, FTIR study confirmed the bonding between PEG molecule and GO sheets (Fig. 2d). The pristine GO membrane showed clear bands at ∼3441 cm^−1^ and 1747 cm^−1^, which is attributed to the O–H and C=O stretching vibration. While C=C band observed at 1651 cm^−1^ and ∼1390 cm^−1^ and ∼1039 cm^−1^ showed presence of C–O functional groups. After modification of GO sheets with PEG, the PGO membranes show almost similar FTIR spectra to pure GO except large intensity, which is due to presence of same functional groups. A broad band at ∼2989 cm^−1^ in PGO membrane appeared due to symmetric and asymmetric stretching modes of methylene group of PEG molecule, which indicated that PEG is successfully crosslinked with GO sheets. While C–O–C and C=O showed strong bands at 1747 cm^−1^. In addition, O–H groups of PGO membranes showed broad bands at 3393 cm^−1^ and 1637 cm^−1^ which is possibly due to that the GO sheets contains OH groups and bound water molecules. These all bands are stronger in intensity than pristine GO membranes. While band at 1109 cm^−1^ was possibly due to the vibration of –C–O– groups from PEG sides.

### 3.2. OSN separation performance

The flux of various solvents through pristine GO and PGO membranes were studied (Fig. 3a). The PGO membrane (350 ± 20 nm) exhibited much higher solvent flux due to its solvent solvated microstructure compare to pristine GO membranes. Fig. 3a and b plots the flux of different solvents *versus* their viscosities through pristine GO and PGO membranes respectively. It can be seen that the flux is decreased with increasing the viscosity of the solvents. Surprisingly, 350 nm-thick PGO membrane shows high flux ∼290 ± 5 L m^−2^ h^−1^ for low viscous hexane (0.31 centipoise cP) as shown in Fig. 3b, which is 10 to 15 times higher than reported GO based membranes (∼18 L m^−2^ h^−1^).^33^ As we increased the viscosity of solvents, the permeance is decreased significantly and membrane showed ∼65 ± 5 L m^−2^ h^−1^ flux for high viscous propanol (2.4 cP) as shown in Fig. 3b. This flux is still higher than reported value for flux for propanol (∼2.5 L m^−2^ h^−1^).^33^ Generally, the flux value is depends on molecular size, and viscosity of solvent in OSN membrane. In addition, the interaction between solvent and membrane is also one of the main factor.^34^ In our research work, the results showed that the viscosity of solvent is major factor for permeance as shown in Fig. 3a. As the viscosity of solvent increased, the flux is significantly decreased. No doubt molecular size and chemical interaction also play their role during permeance.

**Fig. 4:**
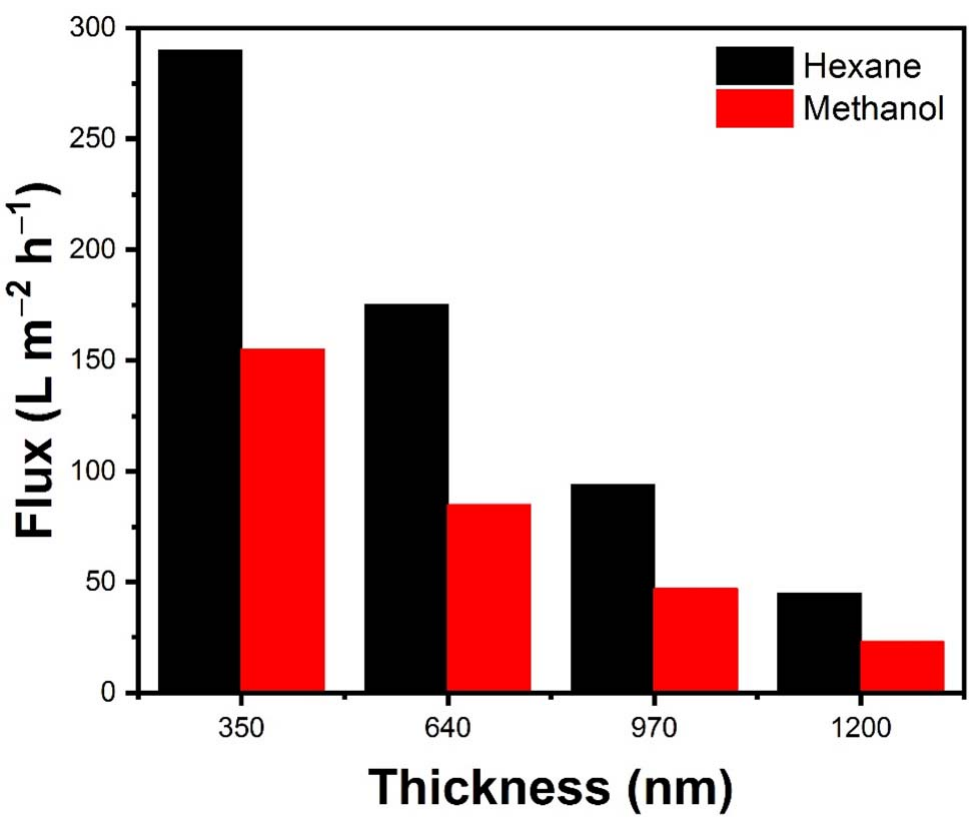
The solvent flux hexane and methanol through PGO membranes with variables thicknesses.

In addition, the solvent flux of PGO membrane strongly depends on the thickness of its coating layers. Generally the membranes with larger thickness show less permeance compared to thin membrane because of low mass transportation.^37^ Then, we studied the solvent flux of PGO membranes with different thickness by using methanol and hexane as solvents (Fig. 4). The solvent flux is decreased, we increased the thicknesses of membranes. This type of trend is very common in GO based membranes which is due to increase in mass transfer resistance. A thicker PGO membrane (1200 ± 20 nm) shows less solvent flux such as ∼45 ± 5 L m^−2^ h^−1^ and ∼23 ± 5 L m^−2^ h^−1^ for hexane and methanol solvents respectively due to its longer tortuous channels.

**Fig. 5:**
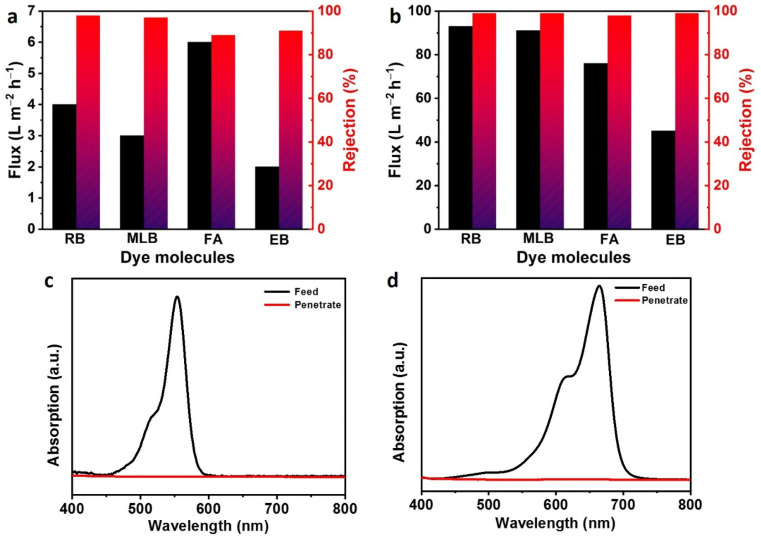
(a and b) Separation performance of pristine 280 nm-thick GO (a) and 350 nm-thick PGO (b) membranes against various dye molecules in methanol solvent. (c and d) Ultraviolet-visible absorption spectra of the feed and permeate of (c) RB, and (d) MLB solution after filtration by PGO membrane.

Further, rejection of probe molecules of both pristine GO and PGO membranes were also evaluated against dye molecules such as methylene blue (MLB), rhodamine B (RB), fuchsin acid (FA), evans blue (EB) in methanol solvent with variable molecular sizes and weights. To ensure the correct assessment of membrane's performance, first we have removed the adsorption effect of GO sheets by stabilizing each test experiment prior to collection of feed, retentate and permeate solution for separation efficiency. Results demonstrated that the flux of PGO membrane is reduced 15 to 20 times compared to pure methanol flux. This reduction in flux is due to the blockage of nanochannels by dye molecules (Fig. 5a and b). First, the pristine GO membrane was used for the separation of dyes with different charge and size diameter in methanol solvent. The 280 ± 20 nm thick GO membrane can only reject dyes up to 97% and showed less permeance less than 7 ± 0.5 L m^−2^ h^−1^ (Fig. 5a). While our fabricated PGO with 350 ± 20 nm thickness showed good separation efficiency > 99% for RB, MLB and EB molecules along high methanol flux of 93 ± 5 L m^−2^ h^−1^ (Fig. 5b). This methanol flux is 20–30 times higher than that of the reported GO membranes.^33,34^ The OSN performance of PGO membrane was also compared with GO based membranes in literature and showed high rejection and flux compared to pristine GO and reported GO membranes so far as show in Table 1. The high rejection of PGO membrane against these probe molecules is due to large size and charge of dye molecule. First, the PGO showed high rejection for large size molecules RB and EB dye (99%), where size is dominant factor. The ultraviolet-visible absorption changes of the methanol solution of RB and MLB dyes after filtration further confirmed the excellent rejection efficiencies (Fig. 5c and d). Second, the charge of dye is also plays major role during separation. The as-prepared PGO membrane is positive in nature and therefore reject cationic dye rapidly due to electrostatic interactions. So the PGO membrane reject cationic MLB dye up to 99% due to positive charge (Fig. 5b).

**Table 1:** OSN performance of PGO membranes compared with other GO based membranes

Membranes	Solvent	Feed solution	Molecular mass (g mol^−1^)	Rejection (%)	Flux (L m^−2^ h^−1^)	Ref.
PGO	Methanol	EB	960	99	45	This work
		MLB	373	99	91	
		RB	479	99	93	
		FA	585.53	98	76	
S-rGO	Methanol	EB	960.8	100	75.3	38
		By	624.5	86.2	76	
		MB	373.9	0	77.2	
		BF	377.8	0	76.9	
HLGO	*n*-Butanol	CG	249	95	2.5	39
	Hexane	CG	249	95	18	
PAN–PA–GO	Ethanol	BB	854	95	1.9	40
GO/COF hybrid	Ethanol	MLB	320	99	51	41
		CR	697	99.82	51	
GO–PPy/PAN	Isopropanol	RB	1017	98.5	3.17	42
GO@PMMA@YSZ	Acetone	MR	269.3	90	7.5	43
GO/CPLI	IPA	RB	479	>94	4.9	44
	DMF			>94	1	
DPAN/PEI–GO–X	Acetone	PEG	<200	96.8	15.7	45
	Ethanol			90	1.5	
	Heptane			90	0.8	

In addition to above discussion, the hydrophobic interaction of dye molecules with PGO membrane also responsible for good separation. Most of dye molecules have benzene rings which can have strong hydrophobic interaction with benzene ring of PGO sheets. This interaction is very common in carbon nanotubes and GO based membranes which effectively separate dye. Therefore, it can be concluded that the size of nanochannels with PGO membranes is approximately of more than 1 nm that the nanochannels size distribution is relatively narrow. Such PGO membrane with superior solvent flux and high separation efficiency should have a great potential for various OSN applications.

### 3.3. Contact angle measurement and stability of PGO membranes

**Fig. 6:**
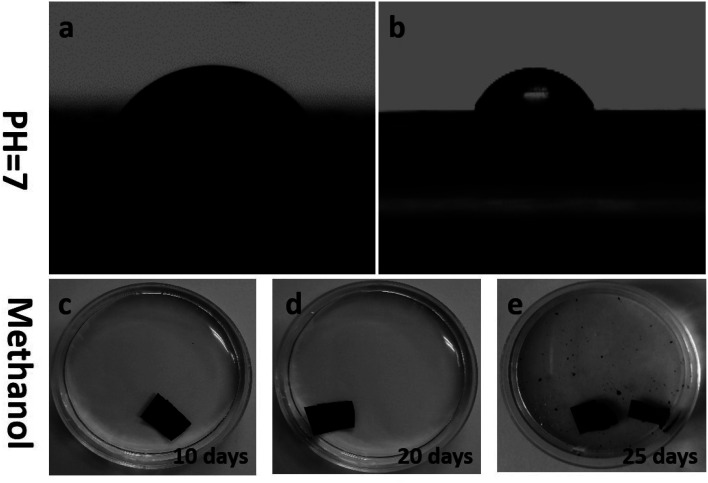
(a and b) The contact angle measurement of GO (a) and PGO (b) membranes. (c–e) Stability of PGO membrane in methanol solvent.

The contact angle measurement of pristine GO and PGO membranes were measured at room temperature and 33% of relative humidity. The Fig. 6a and b exhibited the contact angle of GO and PGO membranes respectively. The PGO membrane exhibited less contact angle ∼45° compared to GO (∼53°) which is due to presence of numerous functional groups in PEG exposed on the surface of membrane.

Further, stability of membrane is very important factor for several separation applications.^46^ As for water desalination, several studies suggested the low stability of GO membranes in water.^47–49^ However, very less works have been reported on stability of GO membranes in organic solvents. Therefore, we studied the chemical stability of freestanding PGO membrane in methanol solvent as shown in Fig. 6c–e. Stability is also one of the important parameter for the practical applications of an OSN membrane.^13^ Our prepared PGO membrane is very stable in methanol and remain its original structure up to 20 days (Fig. 6c and d). However after 20 days, the PGO membrane undergo degradation and completely destroyed within 25 days. This degradation of PGO membrane is possibly due to the presence of remaining functional groups, which easily interact with hydroxyl groups of methanol and cause degradation.

## 4. Conclusion

We have prepared high-performance GO-based membranes for OSN application by cross-linking GO nanosheets with six-armed PEG molecule. As-prepared PEG functionalized GO membrane exhibited a unique layered structure with enhanced interlayer spacing of 1.12 nm. The 350 nm thick PGO membrane exhibits superior separation efficiency (>99%) for MLB, RB and EB dyes, while retaining high flux to methanol solvent. Additionally, PGO membrane is stable up to 20 days in methanol solvent. We hope that this strategy can be useful for fabrication of high-performance membranes based on novel 2D materials for OSN application for large scale applications.

## Conflicts of interest

Authors declare that there is no conflict of interest for this work.

## Supplementary Material
